# A rare association between true thymic hyperplasia and thyroid follicular tumor: a case report

**DOI:** 10.1186/s13256-019-2332-0

**Published:** 2020-01-15

**Authors:** Takumi Kiwaki, Hiroyuki Tanaka, Yutaka Akiyama, Mayumi Akaki, Masaki Tomita, Hiroaki Kataoka

**Affiliations:** 1 Section of Oncopathology and Regenerative Biology, Department of Pathology, Faculty of Medicine, University of Miyazaki, 5200, Kihara, Kiyotake, Miyazaki, 889-1692 Japan; 2 Clinical Laboratory, University of Miyazaki Hospital, Miyazaki, Japan; 3 Department of Thoracic and Breast Surgery, Faculty of Medicine, University of Miyazaki, Miyazaki, Japan

**Keywords:** Thymus, True thymic hyperplasia, Thymic enlargement, Thyroid follicular tumor, Thyroglobulin

## Abstract

**Background:**

True thymic hyperplasia is a rare condition characterized by enlargement of the thymus while its normal structure is retained. True thymic hyperplasia is known to accompany Graves’ disease, but no association between true thymic hyperplasia and thyroid follicular tumor has been reported so far. We report a case of true thymic hyperplasia in a patient with a thyroid follicular tumor.

**Case presentation:**

A 52-year-old Japanese man was referred to our hospital for evaluation of a thyroid mass and a mediastinal mass. His serum thyroglobulin level was high, and hemithyroidectomy was performed to remove the thyroid mass. The resected mass was diagnosed as a follicular tumor of uncertain malignant potential. After resection of the thyroid lesion, the patient’s serum thyroglobulin levels were markedly decreased. Seven months later, the patient underwent resection of the mediastinal mass. On pathological examination, the mass was found to consist of lobules, which formed a corticomedullary structure with Hassall’s bodies, indicating a normal thymic mass with hyperplastic thymic tissue, less organized cellular cords, and intermingled adipose tissue. Immunostaining for cytokeratin 19 and cytokeratin 7 indicated that the lesion was consistent with thymic tissue. The lesion was diagnosed as true thymic hyperplasia, and the histological findings suggested that secondary atrophy had occurred. No evidence of recurrence was observed at 24 months after surgery.

**Conclusions:**

We present a case of a combination of true thymic hyperplasia and thyroidal follicular tumors that, to our knowledge, has not been reported previously. High serum thyroglobulin levels might play a role in hyperplasia of the thymus. Although true thymic hyperplasia is a rare disorder, it should be included in the differential diagnosis of a mediastinal mass in patients with thyroid disease.

## Background

True thymic hyperplasia (TTH), which presents as an enlargement of the thymus, is a rare condition characterized by the growth of the thymic tissue in which the histological structure of the thymic lobule is preserved [1]. It is reported to occur secondary to the chemotherapy of malignant disease [2] or in association with hyperthyroidism [3]. However, the underlying pathogenesis of TTH is largely unknown. We report a case of TTH in a patient with thyroid follicular tumor and discuss the possible etiology of the thymic enlargement. To the best of our knowledge, this type of presentation has not been described previously.

## Case presentation

**Fig. 1:**
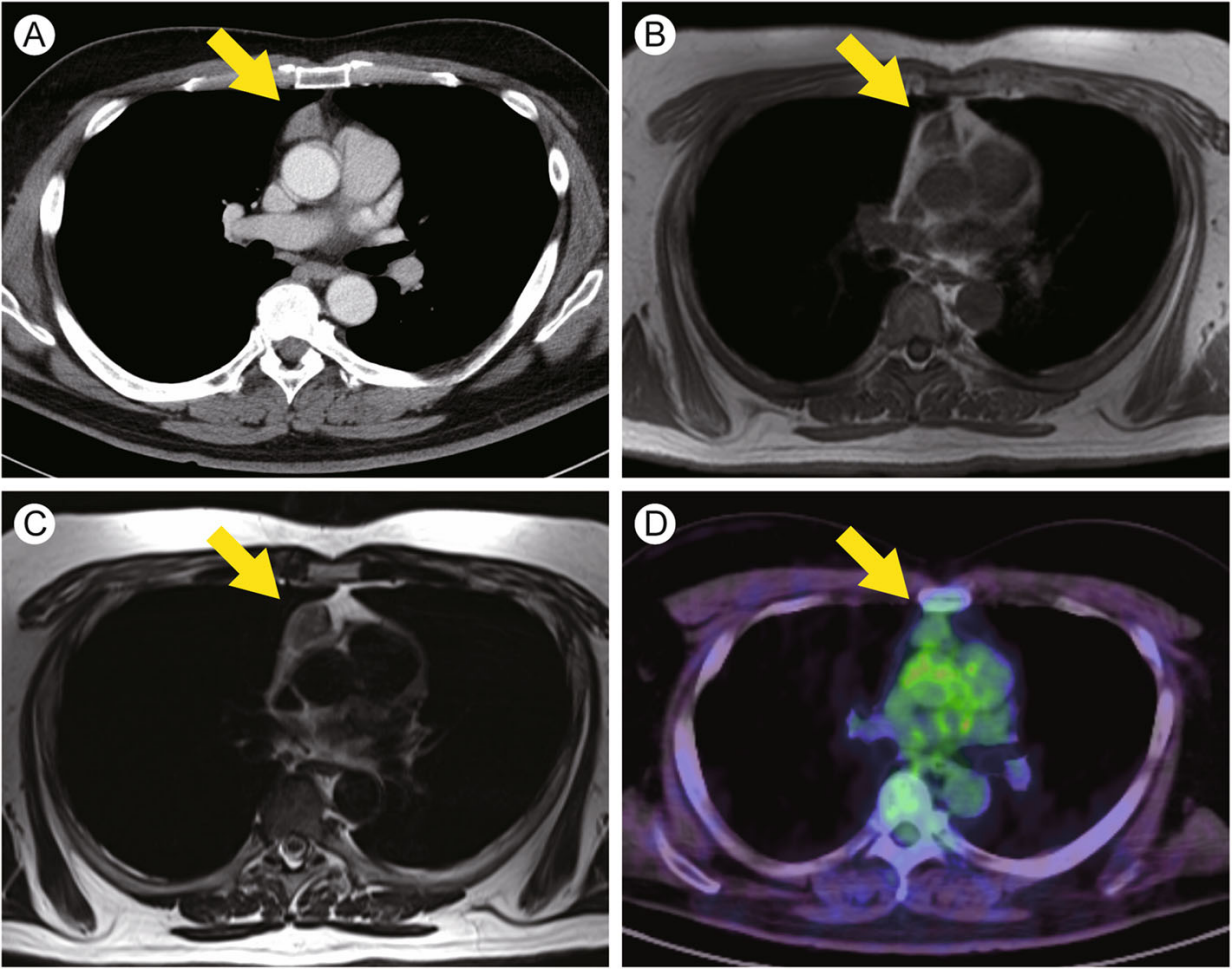
Computed tomography (CT), magnetic resonance imaging (MRI), and positron emission tomography (PET)/CT images of the mediastinal mass (yellow arrow). **a** An enhanced CT scan shows a weakly enhanced mass (size, 6.6 × 2.4 × 2.2 cm). The ratio of the mediastinal mass to the heart was 0.19. **b** and **c** Magnetic resonance imaging T1-weighted (**b**) and T2-weighted (**c**) images reveal areas of isodensity intermingled with high-density areas. **d** PET/CT images demonstrate weak fluorodeoxyglucose uptake

A 52-year-old Japanese man was referred to our hospital for evaluation of a thyroid mass. The patient had a history of hypertension and diabetes. He was taking an angiotensin receptor blocker and a calcium channel blocker for hypertension and a sodium-glucose cotransporter 2 inhibitor for diabetes. The patient had no family history of thyroidal or thymic diseases. The thyroid mass had been discovered incidentally 4 years ago and was found to be benign; the patient was followed up semiannually. After 4 years of follow-up, the hypoechoic area was identified in the mass during an ultrasound examination. Subsequently, the patient was referred to our hospital. He had no symptoms except for neck discomfort. The right lobe of the thyroid was swollen on physical examination. Ultrasonography revealed a relatively homogeneous hypoechoic mass with irregular borders in the right lobe of the thyroid gland. The mass was 6 × 5 cm in size and hypodense in appearance, as seen by computed tomography (CT). Fine-needle aspiration suggested the presence of a follicular lesion. Furthermore, CT revealed the presence of an anterior mediastinal mass approximately 6.6 × 2.4 × 2.2 cm in size (Fig. 1a). Magnetic resonance imaging demonstrated isodensities in the mediastinal mass that were intermingled with high-density areas on both T1- and T2-weighted images, suggesting the presence of adipose tissue (Fig. 1b and c). 2-[^18^F]fluorodeoxyglucose (FDG) positron emission tomography/CT was performed to exclude malignancy, and weak FDG uptake in the mediastinal mass was noted (Fig. 1d). On the basis of these findings, the mediastinal lesion was suspected to be a thymoma. Laboratory tests revealed increased serum levels of thyroglobulin (833 ng/ml), although the levels of free triiodothyronine (T3), free thyroxine (T4), and thyroid-stimulating hormone (TSH) were normal (Table 1). Anti-acetylcholine receptor antibodies were not elevated. No other abnormalities were detected in the serological tests. The patient underwent right hemithyroidectomy, and the pathological diagnosis of the thyroid lesion was a follicular tumor of uncertain malignant potential. Serum thyroglobulin levels were markedly decreased after hemithyroidectomy (7.64 ng/ml). In contrast, the size of the anterior mediastinal mass did not change after surgery.

**Table 1:** Transition of serum thyroid-stimulating hormone, free T3, free T4, and thyroglobulin levels

	Before thyroid operation	After thyroid operation	After thymus operation	Reference range
TSH (μIU/ml)	1.21	3.96	3.30	0.35–4.94
Free T3 (pg/ml)	4.03	2.85	3.32	1.71–3.71
Free T4 (ng/dl)	0.89	1.22	1.10	0.70–1.48
Thyroglobulin (ng/ml)	833	7.64	4.79	−32.7

*TSH* Thyroid-stimulating hormone

The thyroglobulin level was significantly decreased after hemithyroidectomy

**Fig. 2:**
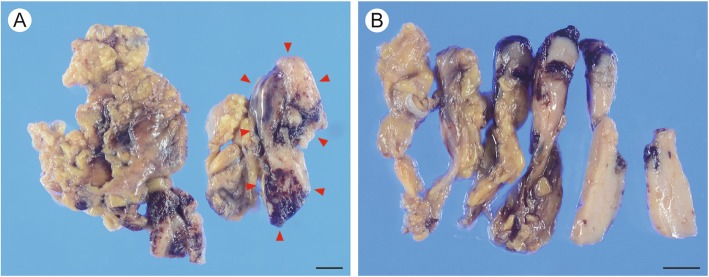
Macroscopic findings of the mediastinal mass after formalin fixation. **a** The resected specimen including the mediastinal mass and surrounding adipose tissue was 12.8 × 7.1 cm in size, and the mediastinal mass (circled with *red arrowhead*) was 6.5 × 2.7 × 1.0 cm in size. **b** The cut surface was yellowish white in color and solid. Bars, 10 mm

**Fig. 3:**
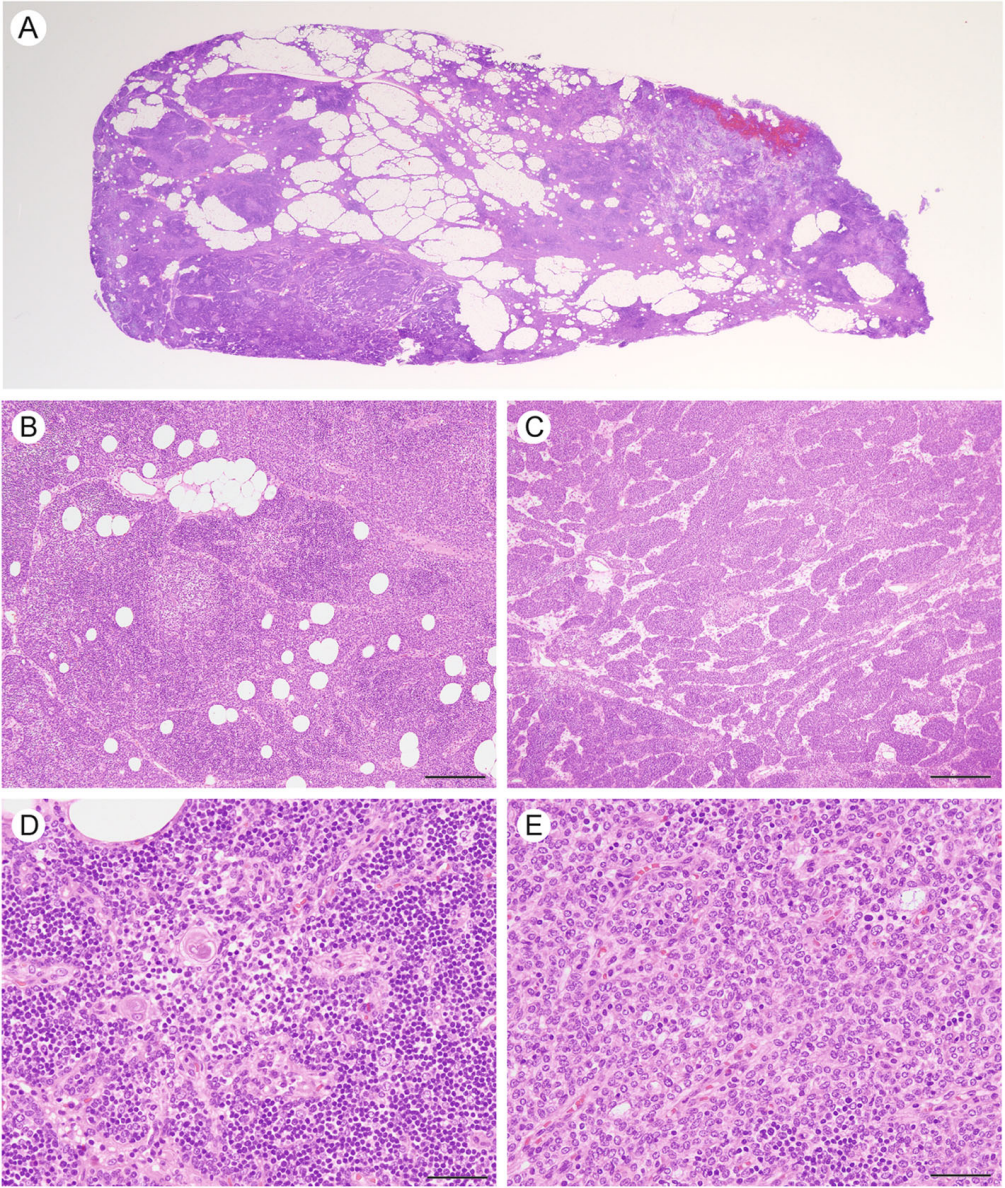
Histology of the mediastinal mass stained with hematoxylin and eosin (H&E). **a** A low-power view shows a nonencapsulated nodule with intermingling adipose tissue elements. **b** Solid portion with large lobular structure. Bar, 500 μm. **c** A portion showing small cellular nests and cords. Bar, 500 μm. **d** Hassall’s bodies. Bar, 100 μm. **e** A representative photo of an epithelial component. Bar, 100 μm

**Fig. 4:**
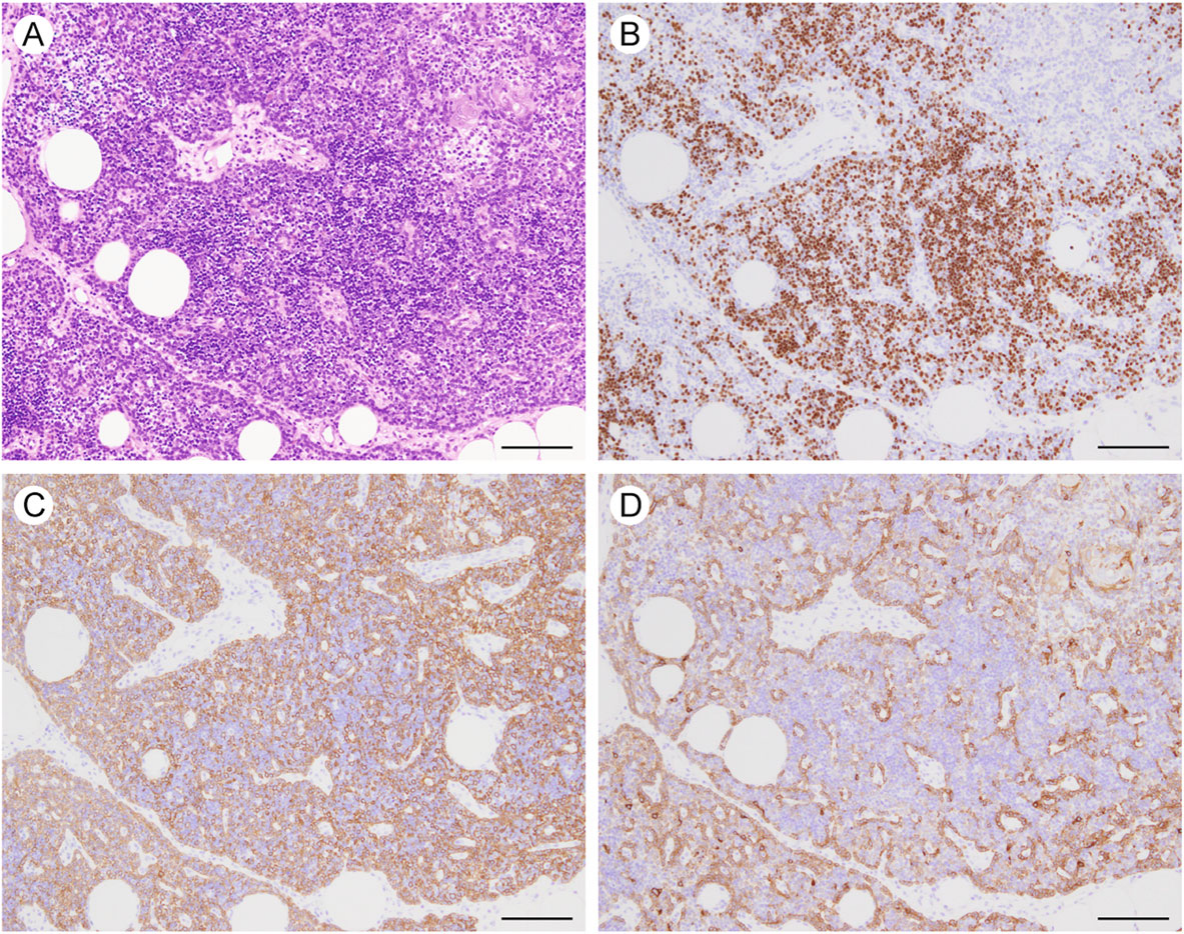
Immunohistochemical findings of the mediastinal mass. **a** hematoxylin and eosin (H&E) stain. **b** Immunostaining for terminal deoxynucleotidyl transferase. **c** Immunostaining for cytokeratin 19 (CK19). **d** Immunostaining for CK7. Bars, 200 μm

Seven months after hemithyroidectomy, the patient underwent thoracoscopic resection of the mediastinal mass. The lesion had not adhered to the adjacent tissue. The resected specimen, which included the mediastinal mass and surrounding adipose tissue, was 12.8 × 7.1 cm in size, and the mass itself was 6.5 × 2.7 × 1.0 cm in size (Fig. 2a). The color of the cut surface was yellowish white (Fig. 2b). Microscopic examination revealed that the lesion was not encapsulated and consisted of solid cellular components intermingled with adipose tissue elements (Fig. 3a). The adipose tissue was predominantly observed in the central portion, whereas the solid cellular components were more commonly observed at the periphery. The cellular components were divided into two histologically distinct portions: a large lobular structure with corticomedullary differentiation resembling normal neonate thymus (Fig. 3b) and cords or small lobular structures separated by loose connective tissue (Fig. 3c). These cellular portions were composed of epithelial cells, lymphocytes, and Hassall’s bodies (Fig. 3d). The epithelial cells had round to oval-shaped nuclei with a fine chromatin pattern, inconspicuous nucleoli, and clear to eosinophilic cytoplasm (Fig. 3e); no apparent monotonous proliferation was observed. The intervening adipose tissues did not show neoplastic changes, and lymphoid follicles with germinal centers were absent. Immunohistochemically, most of the infiltrated lymphocytes were terminal deoxynucleotidyl transferase-positive immature lymphocytes (Fig. 4). The corticomedullary architecture was confirmed using cytokeratin (CK) profiles, as described previously [4], showing CK7 immunoreactivity in the medullary cells but not the cortex cells and CK19 immunoreactivity in the epithelial cells (Fig. 4). The mediastinal lesion was diagnosed as TTH, and no evidence of recurrence was observed 24 months after surgery.

## Discussion and conclusions

Thymic hyperplasia is divided into two distinct conditions: TTH and follicular lymphoid hyperplasia [1]. TTH has been recognized as a complication of chemotherapy for malignant disease and is interpreted as a “rebound” enlargement [2]. This condition has been reported in a patient who received anti-tumor necrosis factor therapy for the treatment of rheumatic disease, in patients treated for human immunodeficiency virus infection, and in a patient following a severe infection [5–7]. Additionally, Graves’ disease has attracted attention as a cause of TTH [3]. However, no reports of an association between TTH and other types of thyroid disease exist in the literature, except for one report on thymic hyperplasia in patients with thyroid cancer [8]. Previously published case reports of TTH with thyroid diseases are summarized in Table 2. To the best of our knowledge, the present case report is the first one to describe TTH in a patient with a thyroid follicular tumor. However, a single case report is insufficient to establish a new association between two diseases, because the possibility of incidental combination cannot be denied. Thus, additional studies are required to evaluate this association between TTH and thyroid follicular tumors.

**Table 2:** Previous case reports of true thymic hyperplasia associated with thyroid diseases

Patient	Age	Sex	Size of thymic mass	Associated thyroid disease	Reference
1	29	M	7.0 × 3.0 cm	Graves’ disease	Budavari *et al.* [3]
2	41	F	Not described	Graves’ disease	Budavari *et al*. [3]
3	24	F	1.5 × 1.0 × 0.8 cm	Graves’ disease	Nakamura *et al.* [9]
4	24	F	Not described	Thyroid cancer	Niendorf *et al*. [8]
4	15	F	Not described	Graves’ disease	Kubicky e*t al*. [10]
5	30	M	6.0 × 5.6 × 2.1 cm	Graves’ disease	Kotwal *et al*. [11]
6	22	F	7.7 × 7.6 × 2.0 cm	Graves’ disease	Song *et al*. [12]
7	49	F	5.5 × 2.4 × 2.1 cm	Graves’ disease	Haider *et al*. [13]
8	24	F	12.4 × 11.0 × 2.5 cm	Graves’ disease	Kennedy *et al*. [14]
Our patient	52	M	6.5 × 2.7 × 1.0 cm	Follicular tumor	–

*M* Male, *F* Female

The mechanism through which Graves’ disease leads to TTH has not yet been elucidated. Two possible mechanisms have been proposed thus far [12]. The first mechanism involves the expression of the TSH receptor in thymic tissue, which mediates thymic overgrowth through an autoimmune response. In some thymic hyperplasia cases accompanied by Graves’ disease, the presence of TSH receptors in the thymic tissues was revealed by a reverse transcription-polymerase chain reaction, northern blot analysis, and immunohistochemistry [9, 15]. The second mechanism involves the induction of hyperplasia in the thymus by the thyroid hormones. Nuclear T3 receptors are expressed in the murine thymic epithelium [16], and thymus enlargement during T3 treatment has been observed [17, 18]. Furthermore, patients with Graves’ disease who underwent radioiodine therapy showed a reduction in thymic volume in parallel with decreased serum T3 levels [19].

In our patient, TSH was not elevated, and no anti-TSH receptor antibodies were detected. Therefore, a TSH-mediated mechanism is unlikely to explain the present observations. Furthermore, T3- or T4-mediated mechanisms are unlikely because neither T3 nor T4 levels were elevated in the patient. In contrast, the serum thyroglobulin level was markedly high due to the thyroid follicular tumor. Elevated thyroglobulin levels are known to be correlated with angiogenesis in the thymus [20]. The resected thymic mass consisted of three different components: neonate-like hyperplastic lobules, completely atrophic adipose tissue, and a relatively hypocellular cord or small nest component. The last component may represent a process of secondary atrophy of the once-enlarged thymus. This phenomenon was described in another study by Kondo *et al.*, and the authors stated that the secondary atrophic mass had finally changed into a thymolipoma [21]. In our patient’s case, we hypothesized that elevated thyroglobulin levels may have contributed to the hyperplasia of the thymus, and spontaneous reduction of thyroglobulin caused by resection of the thyroid follicular tumor may have resulted in secondary atrophy of the tissue. However, currently, we do not have any direct evidence for this association. Further studies will be required to explore the relationship between TTH and thyroglobulin.

Our patient underwent surgical resection of a mediastinal mass, which was diagnosed as TTH after surgery. TTH is a nonneoplastic benign disease. A previous report recommended a conservative approach without surgical intervention for TTH associated with Graves’ disease [13]. To avoid overtreatment, it is necessary to recognize the possibility of TTH in patients with thyroid disease. Evaluation of the radiological features may be useful in excluding a malignant thymic tumor. Compared with thymoma and lymphoma, TTH tends to be located in the midline and shows a quadrilateral, triangular, or bilobed morphology with increased fatty intercalations [22].

In summary, we have presented a case of an unreported combination of TTH and a thyroidal follicular tumor. On the basis of our findings, increased levels of serum thyroglobulin might contribute to thymic hyperplasia. TTH should be included in the differential diagnosis of a mediastinal mass in patients with thyroid disease to avoid overtreatment.

## Data Availability

All data and materials are available from the corresponding author on reasonable request.

## Abbreviations

CK: Cytokeratin
CT: Computed tomography
FDG: Fluorodeoxyglucose
MRI: Magnetic resonance imaging
PET: Positron emission tomography
T3: Triiodothyronine
T4: Thyroxine
TSH: Thyroid-stimulating hormone
TTH: True thymic hyperplasia
